# Comparison of Antibody Responses and Parasite Clearance in Artemisinin Therapeutic Efficacy Studies in the Democratic Republic of Congo and Asia

**DOI:** 10.1093/infdis/jiac232

**Published:** 2022-06-15

**Authors:** Julia C Cutts, Katherine O’Flaherty, Sophie G Zaloumis, Elizabeth A Ashley, Jo Anne Chan, Marie A Onyamboko, Caterina Fanello, Arjen M Dondorp, Nicholas P Day, Aung Pyae Phyo, Mehul Dhorda, Mallika Imwong, Rick M Fairhurst, Pharath Lim, Chanaki Amaratunga, Sasithon Pukrittayakamee, Tran Tinh Hien, Ye Htut, Mayfong Mayxay, M Abdul Faiz, Eizo Takashima, Takafumi Tsuboi, James G Beeson, Francois Nosten, Julie A Simpson, Nicholas J White, Freya J I Fowkes

**Affiliations:** Burnet Institute, Melbourne, Victoria, Australia; Department of Infectious Diseases, University of Melbourne, at the Peter Doherty Institute for Infection and Immunity, Melbourne, Victoria, Australia; Burnet Institute, Melbourne, Victoria, Australia; Centre for Epidemiology and Biostatistics, Melbourne, School of Population and Global Health, University of Melbourne, Melbourne, Australia; Mahidol-Oxford Tropical Medicine Research Unit, Faculty of Tropical Medicine, Mahidol University, Bangkok, Thailand; Centre for Tropical Medicine and Global Health, University of Oxford, Oxford, United Kingdom; Lao-Oxford-Mahosot Hospital-Wellcome Trust-Research Unit, Mahosot Hospital, Vientiane, Lao PDR; Burnet Institute, Melbourne, Victoria, Australia; Department of Infectious Diseases, University of Melbourne, at the Peter Doherty Institute for Infection and Immunity, Melbourne, Victoria, Australia; Department of Immunology, Monash University, Melbourne, Australia; Kinshasa School of Public Health, Kinshasa, Democratic Republic of Congo; Mahidol-Oxford Tropical Medicine Research Unit, Faculty of Tropical Medicine, Mahidol University, Bangkok, Thailand; Centre for Tropical Medicine and Global Health, University of Oxford, Oxford, United Kingdom; Kinshasa School of Public Health, Kinshasa, Democratic Republic of Congo; Mahidol-Oxford Tropical Medicine Research Unit, Faculty of Tropical Medicine, Mahidol University, Bangkok, Thailand; Centre for Tropical Medicine and Global Health, University of Oxford, Oxford, United Kingdom; Mahidol-Oxford Tropical Medicine Research Unit, Faculty of Tropical Medicine, Mahidol University, Bangkok, Thailand; Centre for Tropical Medicine and Global Health, University of Oxford, Oxford, United Kingdom; Myanmar Oxford Clinical Research Unit, Yangon, Myanmar; Mahidol-Oxford Tropical Medicine Research Unit, Faculty of Tropical Medicine, Mahidol University, Bangkok, Thailand; Centre for Tropical Medicine and Global Health, University of Oxford, Oxford, United Kingdom; WorldWide Antimalarial Resistance Network, Asia-Pacific Regional Centre, Bangkok, Thailand; Mahidol-Oxford Tropical Medicine Research Unit, Faculty of Tropical Medicine, Mahidol University, Bangkok, Thailand; Faculty of Tropical Medicine, Mahidol University, Bangkok, Thailand; Laboratory of Malaria and Vector Research, National Institute of Allergy and Infectious Diseases, National Institutes of Health, Rockville, Maryland, USA; Laboratory of Malaria and Vector Research, National Institute of Allergy and Infectious Diseases, National Institutes of Health, Rockville, Maryland, USA; WorldWide Antimalarial Resistance Network, Asia-Pacific Regional Centre, Bangkok, Thailand; Faculty of Tropical Medicine, Mahidol University, Bangkok, Thailand; Mahidol-Oxford Tropical Medicine Research Unit, Faculty of Tropical Medicine, Mahidol University, Bangkok, Thailand; Oxford University Clinical Research Unit, Hospital for Tropical Diseases, Ho Chi Minh City, Vietnam; Department of Medical Research, Ministry of Health and Sports, Yangon, Myanmar; Centre for Tropical Medicine and Global Health, University of Oxford, Oxford, United Kingdom; Lao-Oxford-Mahosot Hospital-Wellcome Trust-Research Unit, Mahosot Hospital, Vientiane, Lao PDR; Institute of Research and Education Development, University of Health Sciences, Vientiane, Lao PDR; Malaria Research Group & Dev Care Foundation, Chittagong, Bangladesh; Division of Malaria Research, Proteo-Science Center, Ehime University, Matsuyama, Japan; Division of Malaria Research, Proteo-Science Center, Ehime University, Matsuyama, Japan; Burnet Institute, Melbourne, Victoria, Australia; Department of Infectious Diseases, University of Melbourne, at the Peter Doherty Institute for Infection and Immunity, Melbourne, Victoria, Australia; Department of Immunology, Monash University, Melbourne, Australia; Mahidol-Oxford Tropical Medicine Research Unit, Faculty of Tropical Medicine, Mahidol University, Bangkok, Thailand; Centre for Tropical Medicine and Global Health, University of Oxford, Oxford, United Kingdom; Shoklo Malaria Research Unit, Mae Sot, Thailand; Centre for Epidemiology and Biostatistics, Melbourne, School of Population and Global Health, University of Melbourne, Melbourne, Australia; Mahidol-Oxford Tropical Medicine Research Unit, Faculty of Tropical Medicine, Mahidol University, Bangkok, Thailand; Centre for Tropical Medicine and Global Health, University of Oxford, Oxford, United Kingdom; Burnet Institute, Melbourne, Victoria, Australia; Centre for Epidemiology and Biostatistics, Melbourne, School of Population and Global Health, University of Melbourne, Melbourne, Australia; Department of Infectious Diseases and Department of Epidemiology and Preventative Medicine, Monash University, Melbourne, Australia

**Keywords:** malaria, antibodies, artemisinin, resistance, parasite

## Abstract

**Background:**

Understanding the effect of immunity on *Plasmodium falciparum* clearance is essential for interpreting therapeutic efficacy studies designed to monitor emergence of artemisinin drug resistance. In low-transmission areas of Southeast Asia, where resistance has emerged, *P. falciparum* antibodies confound parasite clearance measures. However, variation in naturally acquired antibodies across Asian and sub-Saharan African epidemiological contexts and their impact on parasite clearance re yet to be quantified.

**Methods:**

In an artemisinin therapeutic efficacy study, antibodies to 12 pre-erythrocytic and erythrocytic *P. falciparum* antigens were measured in 118 children with uncomplicated *P. falciparum* malaria in the Democratic Republic of Congo (DRC) and compared with responses in patients from Asian sites, described elsewhere.

**Results:**

Parasite clearance half-life was shorter in DRC patients (median, 2 hours) compared with most Asian sites (median, 2–7 hours), but *P. falciparum* antibody levels and seroprevalences were similar. There was no evidence for an association between antibody seropositivity and parasite clearance half-life (mean difference between seronegative and seropositive, −0.14 to +0.40 hour) in DRC patients.

**Conclusions:**

In DRC, where artemisinin remains highly effective, the substantially shorter parasite clearance time compared with Asia was not explained by differences in the *P. falciparum* antibody responses studied.

## Teaser

The substantially shorter *Plasmodium falciparum* parasite clearance time after artemisinin treatment observed in an African site, where artemisinin remains highly effective, compared with Asian sites, cannot be explained by differences in naturally acquired *P. falciparum* antibody responses.

Malaria remains a major cause of disease and death globally, with approximately 241 million cases and 627 000 deaths estimated in 2020, predominantly children living in sub-Saharan Africa [1]. While the scale-up of malaria control interventions, notably long-lasting insecticide nets and access to first-line artemisinin-based combination therapies (ACTs), have led to substantial reductions in the global burden of malaria over the last 2 decades [2, 3], progress has now stalled, and malaria case incidence increased significantly from 2019 to 2020 [1].

Malaria control and elimination is threatened by the emergence of artemisinin-resistant *Plasmodium falciparum*. Artemisinin resistance is characterized by delayed parasite clearance following treatment with artemisinin derivatives. The slow-clearing phenotype is associated with nonsynonymous mutations in the propeller region of the *P. falciparum kelch13* (K13) gene [4, 5]. Currently, in therapeutic efficacy studies, delayed parasite clearance is defined by either a parasite clearance half-life (PC_½_) of ≥5 hours or persistent parasitemia by microscopy on day 3 after treatment [6]. Reports of artemisinin resistance first emerged in Western Cambodia in 2009, and over the following decade therapeutic efficacy studies revealed that artemisinin resistance continued to spread throughout the Greater Mekong subregion [5, 7–10] and west to West Bengal in India [11]. In African countries, despite a high diversity of nonsynonymous mutations present in *kelch13* in *P. falciparum* parasite*s,* the gene is not currently undergoing strong selection [12, 13]. More recently, however, de novo emergence of *kelch13* mutations conferring resistance were identified in Rwandan and Ugandan patients [14–16]. If resistance becomes widespread in high-burden countries in Sub-Saharan Africa, it is estimated that ACT treatment failure could lead to an excess of 78 million cases and 116 000 deaths over a 5-year period [17]. It is therefore essential that the efficacy of artemisinin is accurately monitored to inform appropriate public health responses to prevent or contain the emergence and spread of artemisinin resistance in Africa and to preserve the life span of artemisinin derivatives.

While detection of artemisinin-resistant polymorphisms in *P. falciparum* infections is unequivocal, parasite clearance rates in response to ACT vary considerably between patients. Many factors contribute to this variability, including differences in splenic function, pharmacokinetics, parasite factors including the stage and synchronicity of the infection, and naturally acquired immunity [18–21]. Immunity to clinical malaria develops after repeated infections and predominantly targets the blood stages of malaria parasites [22]. It develops more quickly in high-transmission areas [23, 24], and it influences the epidemiology of malaria. In high-transmission areas such as sub-Saharan Africa, children bear the majority of the burden of symptomatic disease, whereas in low-transmission areas all ages are susceptible. Naturally acquired immunity is associated with reduced parasite densities [25], thus in higher-transmission settings immunity may mask the emergence of slow-clearing phenotypes, by reducing baseline parasitemia (a risk factor for delayed clearance) [20] or enhancing parasite clearance after treatment [26, 27]. Understanding the potential confounding effect of immunity on parasite clearance measures across a range of epidemiological settings is important to inform artemisinin resistance surveillance.

In a multinational therapeutic efficacy study (Tracking Resistance to Artemisinin Collaboration [TRAC]), we found that naturally acquired antibodies to *P. falciparum* varied within- and between-study sites in Southeast Asia, a region of relatively low malaria transmission where adults experience the greatest malaria burden, and antibody responses were associated with shorter PC_½_ following artemisinin treatment [26–28]. How immunity differs across the markedly different epidemiological contexts of Southeast Asia and sub-Saharan Africa is unknown, along with its impact on parasite clearance in therapeutic efficacy studies. Given the public health consequences of the emergence of artemisinin resistance in sub-Saharan Africa, we investigated antibody responses to selected *P. falciparum* antigens and artemisinin resistance indicators in children with acute uncomplicated malaria enrolled in the TRAC study in Kinshasa, Democratic Republic of Congo (DRC), an area of high perennial transmission. We compared these with results in the TRAC Asian sites, where malaria transmission is comparably low and heterogenous.

## METHODS

### Study Design and Procedures

Plasma samples were taken from 118 patients with uncomplicated falciparum malaria (of a total of 119) from Kinshasa, DRC, participating in the TRAC multicenter open-label drug-efficacy randomized control trial (Clinical Trials Registration NCT01350856). Antibody responses were compared with those in patients from Asian sites from the same trial, which have been published elsewhere [26]. Details of the original study design, inclusion criteria and procedures, as well as characterization of antibody responses in patients from Asian sites have been published elsewhere [5, 26]. Informed consent was obtained from all patients, and ethical approval was granted by the Oxford Tropical Research Ethics Committee (06/11), Alfred Hospital Committee for Ethics, Australia (485/12) the institutional review board, national ethics committee, or both for each site. Patients in the DRC were admitted to the hospital for supervised treatment with either artemether-lumefantrine or artesunate monotherapy at a dose of 4 mg/kg/d for 3 days, followed by a full course of artemether-lumefantrine [5]. Body temperature, hematocrit, and blood smears were obtained for malaria parasite counts at 0, 4, 6, 8, and 12 hours and then every 6 hours until 2 consecutive counts were negative. The full sequence of *kelch13* was determined for isolates collected at admission [5]. Herein, *kelch13* mutations conferring resistance were defined as mutations after position 440. Patients infected with these parasites were compared with those with either wild-type infections or infections with *kelch13* mutations before amino acid position 441.

### Measurement of Immunoglobulin G Antibodies to Parasite Antigens

At enrollment, plasma concentrations of total antigen-specific immunoglobulin (Ig) G were measured by enzyme-linked immunosorbent assay (ELISA), as described elsewhere [26]; recombinant *P. falciparum* merozoite antigens [29], including merozoite surface protein (MSP) 1, C-terminal 19-kDa region (MSP1_19_), MSP2, FC27 allele (MSP2_FC27_); MSP2, 3D7 allele (MSP2_3D7_); MSP3; MSP6; MSP7; apical membrane antigen 1, reticulocyte-binding ligand homologue 2 (Rh2), erythrocyte-binding antigen (EBA) 175, region 2 (EBA175_RII_), EBA175, regions 3–5 (EBA175_RIII–V_), and circumsporozoite protein (CSP) [30], were measured with high-throughput ELISA, as described elsewhere [26, 27]. All steps were performed on a Janus high-throughput liquid handling platform (Perkin Elmer), except for antigen coating. Antibody positivity was defined as optical density values higher than 2 standard deviations above the mean of nonexposed Melbourne controls. Antibody levels to variant surface antigens (VSAs) of *P. falciparum*-infected erythrocytes (3D7 strain) at the pigmented trophozoite stage were measured with flow cytometry [31].

### Statistical Analysis

To investigate the association between antibodies and parasite clearance, the outcome variable PC_½_ (in hours) was derived using the WWARN parasite clearance estimator [19]. Multiple linear regression analysis was performed to determine the mean difference in PC_½_ between antibody response (seropositive/seronegative and continuous) for each antibody, adjusting for age and for whether patients had received artemether-lumefantrine or initial artesunate monotherapy. Median antibody levels and the proportion of IgG responders were compared for the DRC and Asian sites.

## RESULTS

### Patient Characteristics, Artemisinin Resistance, and *P. falciparum* Antibody Profiles

A total of 118 patients with uncomplicated falciparum malaria from Kinshasa, DRC, were included in the current study. All patients were children (median age [interquartile range (IQR)], 5 [3–6] years [3, 6]; 63 of 118 [53.4%] male), differing from patients enrolled in the Asian study sites, who were typically men of working age (Supplementary Table 1). At enrollment, the median (IQR) *P. falciparum* density in patients from DRC was 60 037/µL (IQR, 35 042–109 900/µL), which was similar to the median parasite densities in patients from Asian sites (Supplementary Table 1). In patients from Kinshasa, the median PC_½_ (IQR) was 2 (1.6–2.4) hours, with only 2 patients having a PC_½_ ≥5 hours and detectable parasitemia at day 3 and PC_½_ differed by treatment group (2.2 [1.7–2.5] hours for artemether-lumefantrine vs 1.85 [1.3–2.2] hours for artesunate [4mg/kg]).

The prevalence of *kelch13* mutations in the propellor region was 2.5% (3 of 118), but none of these 3 children had PC_½_ ≥ 5 hours, and these markers (A578S, Q613E, and S522C) have not been associated with resistance elsewhere [32]. With the exception of patients in Ramu, Bangladesh, and Attapeu, Laos (≤2.6 hours), patients from most of the Asian sites had a higher median PC_½_ (range, 3–6.95 hours) and higher prevalence of *kelch13* mutations (range, 9.1%–60.4%) (except for those from Ramu, Bangladesh; Attapeu, Laos; and Ratanakiri, Cambodia [≤0.8%]) (Supplementary Table 1**)** [5, 26].

**Figure 1.**
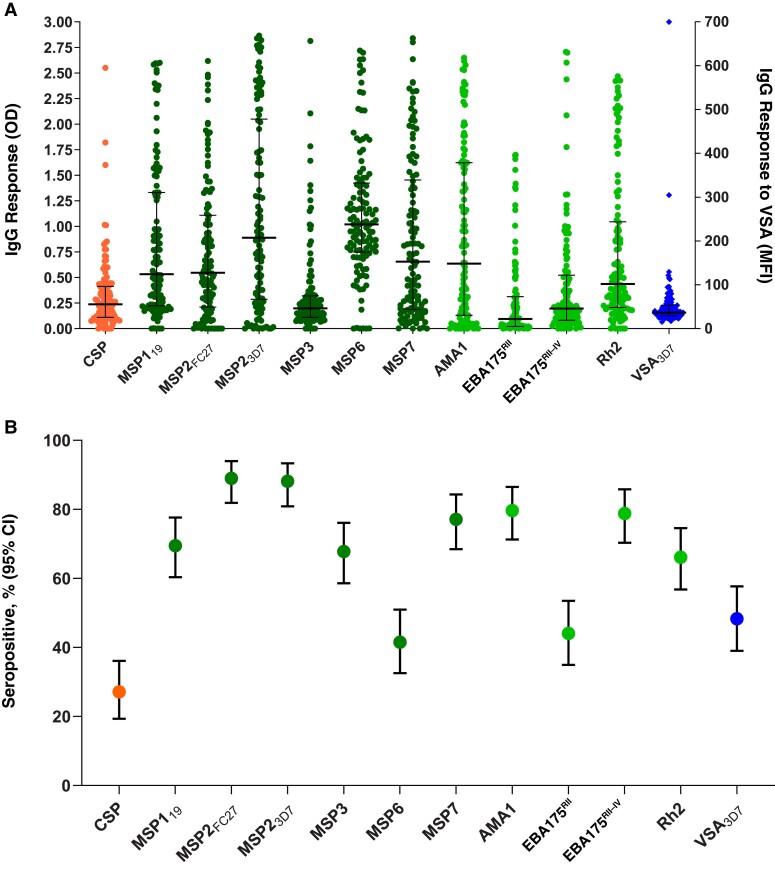
Immunoglobulin (Ig) G responses to *Plasmodium falciparum* antigens in the Tracking Resistance to Artemisinin Collaboration (TRAC) study participants from Kinshasa, Democratic Republic of Congo. *A*, Dots represent individual IgG responses to selected recombinant *P. falciparum* antigens; black horizontal lines, medians; and whiskers, interquartile ranges. Abbreviation: MFI, mean flourescence intensity; OD, optical density. *B*, Seroprevalences of IgG antibody responses to each antigen. Sporozoite antigen is shown in orange, merozoite surface proteins (MSPs) in dark green and rhoptry/microneme proteins in light green (measured by enzyme-linked immunosorbent assay), and variant surface antigens on 3D7-infected erythrocytes in blue (measured by flow cytometry). Seropositivity cutoffs for each antigen were as follows: circumsporozoite protein (CSP), 0.40; MSP1_19_, 0.27; MSP2_FC27,_ 0.03; MSP2_3D7_, 0.07; MSP3, 0.14; MSP6, 1.15; MSP7, 0.17; apical membrane antigen 1 (AMA1), 0.07; erythrocyte-binding antigen (EBA) 175, region 2 (EBA175_RII_), 0.11; EBA 175, regions 3–5, EBA175_RIII-,_ 0.07; reticulocyte-binding ligand homologue 2 (Rh2), 0.30; and VSA 3D7 336.1. Abbreviation: CI, confidence interval.

IgG responses to multiple *P. falciparum* blood-stage antigens (merozoite and infected RBC) and the sporozoite antigen CSP, were quantified at enrollment, before antimalarial treatment (Figure 1). Both the levels and seroprevalence (27.1% [32 of 118]) of IgG responses to the sporozoite antigen CSP were lower in patients from Kinshasa (Supplementary Figure 1 and Supplementary Table 2), compared with the Asian sites (Supplementary Table 2**).** In patients from Kinshasa, seroprevalences of IgG responses to blood-stage antigens (MSP1_19_, apical membrane antigen 1, MSP2_FC27_, MSP2_3D7_, MSP3, MSP6, MSP7, EBA175_RII_, EBA175_RIII–V_, Rh2, and variant surface antigen, 3D7 strain (VSA_3D7_) ranged from 41.5% (49 of 118) to 89.0% (105 of 118). Both levels and seroprevalences to these antigens were broadly similar to responses in the Asian sites (Supplementary Figure 1 and Supplementary Table 2).

### Associations Between Antibody Responses and Treatment Efficacy

We have previously reported that antibody responses are associated with shorter PC_½_ in Asian TRAC sites (mean difference in PC_½_ according to seropositivity, −0.16 to −0.65 hours overall and −0.07 to −0.52 hours in those with wild-type infections, depending on antigen) [26]. In contrast, there was no evidence for an association between antibody seropositivity or level for antibodies to *P. falciparum* antigens and shorter PC_½_ in patients from Kinshasa, DRC, after adjusting for age and treatment group (Table 1 and Supplementary Table 3 respectively). The mean PC_½_ for patients who were seronegative for antibodies to each of the *P. falciparum* antigens studies ranged from 1.91 to 2.15 hours, which was similar to the mean PC_½_ in seropositive patients (mean difference in PC_½,_ close to zero) (Table 1). Similarly, there was no association between antibody levels and PC_½_ when antibody responses were analyzed as continuous variables; for every 2-fold increase in IgG level (optical density value), the change in PC_½_ was also close to zero (range, −0.07 to 0.15 hours) (Supplementary Table 3). Sensitivity analysis whereby the 3 cases with *kelch13* mutants conferring resistance were removed (all of which had PC_½_ <5 hours) did not significantly alter results.

**Table 1.** Associations Between Antibody Seropositivity and Parasite Clearance Half-Life in 118 Patients from Kinshasa, Democratic Republic of Congo, With Adjustment for Age and Treatment^a^

Antigen	No. (%) Seropositive	PC_½_ in Seronegative Patients, Mean, h^b^	Difference in PC_½_ if Seropositive, Mean (95% CI), h	*P* Value
Transmission-stage antigen				
CSP	32 (27.1)	2.10	−.11 (−.48 to .26)	.56
Merozoite antigens				
MSP1_19_	82 (69.5)	2.14	−.09 (−.44 to .27)	.64
MSP2_FC27_	105 (89.0)	2.15	−.10 (−.63 to .44)	.72
MSP2_3D7_	104 (88.1)	2.05	−.002 (−.53 to .52)	>.99
MSP3	80 (67.8)	2.02	−.11 (−.25 to .46)	.56
MSP6	49 (41.5)	2.13	−.14 (−.47 to .20)	.43
MSP7	91 (77.1)	2.07	−.01 (−.40 to .42)	.95
AMA1	94 (79.7)	2.05	−.03 (−.40 to .45)	.91
EBA175_RII_	52 (44.1)	1.91	.40 (.05–.75)	.03
EBA175_RIII–V_	93 (78.8)	2.03	−.02 (−.40 to .44)	.93
Rh2	78 (66.1)	1.95	.17 (−.19 to .53)	.35
Variant surface antigens				
VSA_3D7_	57 (48.3)	2.01	.14 (−.22 to .51)	.44

Abbreviations: AMA1, apical membrane antigen 1; CI, confidence interval; CSP, circumsporozoite protein; EBA175_RII_, erythrocyte-binding antigen 175, region 2; EBA175_RIII–V_, erythrocyte-binding antigen 175, regions 3–5; MSP, merozoite surface protein; PC_½_, parasite clearance half-life; Rh2, reticulocyte-binding ligand homologue 2; VSA_3D7_, variant surface antigen, 3D7 strain

a Linear regression analysis adjusted for age and treatment arm. Analysis did not adjust for *kelch13* genotype because only 3 individuals had *Plasmodium falciparum* infections with *kelch13* mutations associated with resistance. Sensitivity analysis indicated their inclusion had no effect on the association between antibody positivity and PC_½_.

b In patients with a mean age of 4.6 years receiving 4 mg/kg/d of artesunate for 3 days (base values for linear regression model).

## DISCUSSION

In the context of a multinational therapeutic efficacy study, we found that children with uncomplicated malaria from Kinshasa, DRC, had levels of antibodies to selected *P. falciparum* antigens that were broadly comparable to those seen in patients from Asian TRAC sites. In contrast to what was previously reported for pooled individual patient data from the TRAC sites in Asia [26], these antibody responses were not associated with faster parasite clearance. Thus, the substantially more rapid *P. falciparum* parasite clearance observed in Kinshasa compared with Asia was not explained by differences in the concentrations of the *P. falciparum* antibodies measured in this study. This may be because artesunate was still highly effective at killing drug-sensitive ring-stage parasites in DRC and mechanisms independent of the antibody-responses examined were sufficient for rapid parasite clearance.

Naturally acquired immunity to *P. falciparum* malaria, which protects against high-density infections and clinical symptoms, is acquired after repeated infections and underpins the age-dependent distribution of clinical disease in malaria-endemic areas [22]. Reflecting this, patients with uncomplicated clinical malaria enrolled in the TRAC study were young children in Kinshasa, DRC, an area of high perennial transmission, whereas in relatively low-transmission countries of Asia, patients were typically adults, a high-risk group in this region due to occupational exposure [33, 34]. Despite the different age profiles of the African and Asian participants, all infections had progressed to acute, uncomplicated clinical disease and all met the same study inclusion criteria; therefore, by definition, both groups lacked sufficient disease controlling activity for that infection. In these patients, children from DRC had levels of antibodies to relatively conserved merozoite antigens that were comparable to those of patients from Asian sites, who were mostly adults, suggesting that they may have had similar cumulative exposure.

While we assessed total IgG responses specific for a range of *P. falciparum* antigens, including relatively conserved antigens and those genetically diverse across parasite populations, considerable variation was observed in responses to individual antigens both within and across populations and the potential role of functional antibody responses was not investigated. Functional antibody responses, including parasite opsonization to promote phagocytosis and antigen-specific deposition of complement factors were associated with significantly reduced PC_½_ estimates in patients recruited from Asian study sites at magnitudes comparable to or greater than measured total IgG responses [26, 27]. Therefore, further research into the most appropriate antibody biomarkers for use in future therapeutic efficacy studies in the African context is warranted.

In contrast to our previous findings in pooled Asian patients [26], we found that despite comparable levels of antibodies to the *P. falciparum* antigens investigated, there was no evidence for an association between these antibody responses and PC_½_ among children from Kinshasa, DRC, receiving either artesunate or artemether-lumefantrine. This may be because artemisinin derivatives are so effective in clearing wild-type parasites through immune-independent mechanisms, such as “pitting,” whereby damaged drug-sensitive wild-type ring-stage parasites are removed from the infected erythrocyte by the spleen, that any additional antibody-mediated effect is minimal [35–39]. In contrast, *kelch13*-mutant ring-stage parasites that survive exposure to artemisinin derivatives develop into trophozoites, schizonts, and merozoites [40], which are then subject to antibody-dependent immune clearance mechanisms, including opsonic phagocytosis and merozoite lysis [41, 42].

Consistent with this hypothesis, we previously reported that among adults from the Asian TRAC sites, the magnitude of effect of antibody positivity on PC_½_ was larger in patients infected with parasites containing *kelch13* mutations conferring resistance than in those with wild-type parasites or parasites with other *kelch13* mutations. However, the association between antibodies and PC_½_ was still evident in Asian patients with wild-type parasites, unlike what was observed in DRC patients [26]. This may be related to the younger age of the African patients. In Malian children receiving oral artesunate for treatment of uncomplicated malaria, antibody responses to autologous infected erythrocytes were positively correlated with age and negatively correlated with peak pitting rate [39, 43]. This immune-independent parasite clearance mechanism may partially explain why, overall, DRC patients had substantially more rapid *P. falciparum* parasite clearance than Asian patients, despite some children having been treated with artemether-lumefantrine, which was associated with longer parasite clearance time (median, 2.2 hours vs 1.85 hours with artesunate [5]). Differences in innate immune mechanisms of parasite removal in children relative to adults [44, 45] may also play a role in the faster parasite clearance observed in Kinshasa compared with Asia. Further investigation is required to elucidate the factors underpinning differences in PC_½_ between populations, but our findings suggest that naturally acquired antibodies to the *P. falciparum* antigens investigated here do not contribute to differences in PC_½_ between patients from the Kinshasa, DRC, and Asian study sites.

Since the TRAC study was completed in 2013, artemisinin resistance has continued to spread throughout the Greater Mekong subregion [9, 10], west to West Bengal in India [11] and resistance to piperaquine and other partner drugs has emerged and spread in the Greater Mekong subregion [9]. Nonsynonymous mutations in *kelch13* have been identified in isolates from west, central and east Africa [13] and de novo emergence of *kelch13* mutations conferring slow-clearance resistance phenotypes have been identified in Rwandan (R561H) [14, 15] and Ugandan patients (A675V or C469Y) [16]. In populations living in highly endemic areas in Africa, with naturally acquired immunity, the current PC_½_ cutoff for defining resistance (≥5 hours) may be too strict [16]. As the artemisinin resistance evolves in Africa, it will be important to define how to quantitate the contribution of immunity to parasite clearance and its confounding effects on measures of parasite clearance in children, who form the sentinel surveillance populations for artemisinin therapeutic efficacy studies. Current threshold measures of resistance, including day 3 parasite positivity and PC_½_ of ≥5 hours, need to be revised to facilitate monitoring of the emergence of artemisinin resistance across Africa.

## Supplementary Data

Supplementary materials are available at *The Journal of Infectious Diseases* online (http://jid.oxfordjournals.org/). Supplementary materials consist of data provided by the author that are published to benefit the reader. The posted materials are not copyedited. The contents of all supplementary data are the sole responsibility of the authors. Questions or messages regarding errors should be addressed to the author.

## Notes

***Acknowledgments.*** We thank the following: all the patients, for their participation in these studies; Tracking Resistance to Artemisinin Collaboration (TRAC) study investigators; Annie Mo (National Institutes of Health), for providing erythrocyte-binding antigen 175, region 2; Robin Anders (La Trobe University), for providing MSP2; and Alistair McLean, for technical support.

***Disclaimer.*** The funders had no role in study design, data collection and analysis, the decision to publish, or the preparation of the manuscript.

***Financial support***. This work was supported by the National Health and Medical Research Council of Australia (NHMRC) (project grant 1060785 to F. N., J. A. S., and F. J. I. F., program grant 637406 to J. G. B., and career development fellowship 1166753 to F.J.I.F., and leadership investigator grants 1077636 to J. G. B. and 1196068 to J.A.S.); the Australian Research Council (Future Fellowships FT130101122 to F. J. I. F. and FT0992317 to J. G. B.); the Ramaciotti Foundation (establishment grant 3245/2011 to F. J. I. F.); the Ian Potter Foundation (grant to F. J. I. F.); the Australian Centre for Research Excellence on Malaria Elimination, funded by the NHMRC of Australia (grant 1134989); the Victorian Operational Infrastructure Support Program (support to the Burnet Institute); the UK Department for International Development; the Worldwide Antimalarial Resistance Network; the Intramural Research Program of the National Institute of Allergy and Infectious Diseases, National Institutes of Health; and the Wellcome Trust (grant 220211). Funding to pay the Open Access publication charges for this article was provided by the Burnet Institute.

***Potential conflicts of interest.*** All authors: No reported conflicts. All authors have submitted the ICMJE Form for Disclosure of Potential Conflicts of Interest. Conflicts that the editors consider relevant to the content of the manuscript have been disclosed.

## Supplementary Material

jiac232_Supplementary_Data
