# Supplementary material for: Comparison of Antibody Responses and Parasite Clearance in Artemisinin Therapeutic Efficacy Studies in the Democratic Republic of Congo and Asia
Source: J Infect Dis. 2022 Jun 15;226(2):324–31. doi: 10.1093/infdis/jiac232 (PMC9400417; doi:10.1093/infdis/jiac232)
Supplement: jiac232_Supplementary_Data [file jiac232_supplementary_data.docx]

Supplementary Table 1: Baseline demographic characteristics of study participants by study site

| Country and study site | N | Male, (N (%)) | Age, years (median [25^th^-75^th^ percentiles], range) | | Initial parasite density (parasites/µL), median,  [25^th^-75^th^ percentiles], range | | | | Treatment arms | | PC_1/2_ hours, median, [25^th^-75^th^ percentiles], range | PC_1/2_ ≥ 5 hours, n/N (%) | Parasitaemia at day 3, n/N (%) | *Kelch13* mutants^a^, n/N (%) | | PC_1/2_ ≥ 5 hours **and** *Kelch13* mutants^a^, n/N (%) | |
| --- | --- | --- | --- | --- | --- | --- | --- | --- | --- | --- | --- | --- | --- | --- | --- | --- | --- |
| Bangladesh |  |  |  | |  | | | |  | |  |  |  |  | |  | |
| Ramu | 49 | 42 (85.7) | 26 [20-35],  10-55 | | 32154 [19594-50868],  10048-224196 | | | | AS2 or AS4 | | 2.6 [2-3.2], 0.7-5.4 | 1/49 (2) | 1/48 (2.1) | 0/49 (0) | | 0/49 (0) | |
| Cambodia |  |  |  | |  | | | |  | |  |  |  |  | |  | |
| Pailin | 96 | 83 (86.5) | 24.5 [19-37.5], 10-56 | | 45216 [26062-87857],  3712-389988 | | | | AS4 | | 6.1 [4.9-7.2], 2.4- 9 | 69/96 (71.9) | 71/96 (74.0) | 77/96 (80.2) | | 58/96 (60.4) | |
| Preah Vihear | 120 | 82 (68.3) | 20 [14-29],  4-58 | | 56583 [42704-86162],  13942-311237 | | | | AS2 or AS4 | | 3 [2.5-4.2], 1.2-12.6 | 26/120 (21.7) | 29//120 (24.2) | 22/113 (19.5) | | 19/119 (16.0) | |
| Pursat | 119 | 108 (90.8) | 25 [19-33.5], 3-60 | | 56520 [35168-107765],  9797-284861 | | | | AS4 | | 5.6 [4.3-6.7], 1.7-11.8 | 72/119 (60.5) | 85/119 (71.4) | 76/115 (66.1) | | 61/118 (51.7) | |
| Ratanakiri | 120 | 78 (65.0) | 14 [9-19.5],  2-55 | | 62109 [32844-94702],  5024-310860 | | | | AS2 or AS4 | | 3 [2.3-3.5], 0.7- 8.8 | 5/120 (4.2) | 11/118 (9.3) | 4/116 (3.5) | | 1/120 (0.8) | |
| DRC |  |  |  | |  | | | |  | |  |  |  |  | |  | |
| Kinshasa | 118 | 63 (53.4) | 5 [3-6],  0.73-8 | | 60037 [35042-109900],  11555-605329 | | | | AL or AS4 | | 2 [1.6-2.4], 0.7-7^b^ | 2/118 (1.7) | 2/118 (1.7) | 3/118 (2.5) | | 0/118 (0) | |
| Laos |  |  |  | |  | | | |  | |  |  |  |  | |  | |
| Attapeu | 85 | 57 (67.1) | 23 [13-29],  6-60 | | 51496 [28637-90432],  12811-198574 | | | | AS2 or AS4 | | 2 [1.6-2.7], 1.1- 9.2 | 5/84 (6.0) | 10/83 (12.1) | 3/84 (3.4) | | 2/84 (2.4) | |
| Myanmar |  |  |  | |  | | | |  | |  |  |  |  | |  | |
| Shwe Kyin | 77 | 64 (83.1) | 24 [19-31],  13-54 | | 64307 [27088-115552],  10640-420006 | | | | AS2 or AS4 | | 3.1 [2.6-4.1], 1.3-8.6 | 9/77 (11.7) | 12/77 (15.6) | 17/76 (22.4) | | 7/77 (9.1) | |
| Thailand |  |  |  | |  | | | |  | |  |  |  |  | |  | |
| Mae Sot | 117 | 92 (78.6) | 29 [23-37],  18-58 | | 37492 [17584-83273],  2560-327062 | | | | AS2 or AS4 | | 4.9 [3.7-6.4], 0.6-10.1 | 55/117 (47.0) | 53/117 (45.3) | 59/114 (51.8) | | 37/116 (31.9) | |
| Ranong | 22 | 16 (72.7) | 32 [26-39],  19-53 | | 45656 [24618-80384],  5903-94451 | | | | AS2 or AS4 | | 5.3 [3.5-6.4], 2.4-13.8 | 13/22 (59.1) | 12/20 (60.0) | 13/20 (65.00) | | 12/22 (54.6) | |
| Srisaket | 36 | 36 (100) | 28 [22-39],  16-54 | | 28134 [13000-75234],  4346-192997 | | | | AS4 | | 7.0 [4.3- 8.7], 1.6-13.9 | 24/36 (66.7) | 23/34 (67.7) | 29/35 (82.9) | | 20/35 (57.1) | |
| Vietnam |  |  |  | |  | | | |  | |  |  |  |  | |  | |
| Binh Phuoc | 118 | 91 (77.1) | 26 [19-39],  4-61 | | 49738 [23864-96084],  9797-205230 | | | | AS2 or AS4 | | 3.1 [1.9-5.3], 0.7-8.9 | 33/118 (28.0) | 38/118 (32.2) | 28/116 (24.1) | | 24/118 (20.3) | |
|  | | | |  | |  |  |  | |  | | | | |  | |  |

^a^Non-synonymous SNPs in *Kelch13* after position 440

^b^PC_1/2_ hours (median, [25th-75th percentiles], range) differed by treatment group: AL: 2.2 [1.7-2.5], 1.2-4.6 versus AS4: 1.85 [1.3-2.2], 0.7-7

IQR, interquartile range; R, range; PC_1/2_, Parasite clearance half time (hours)


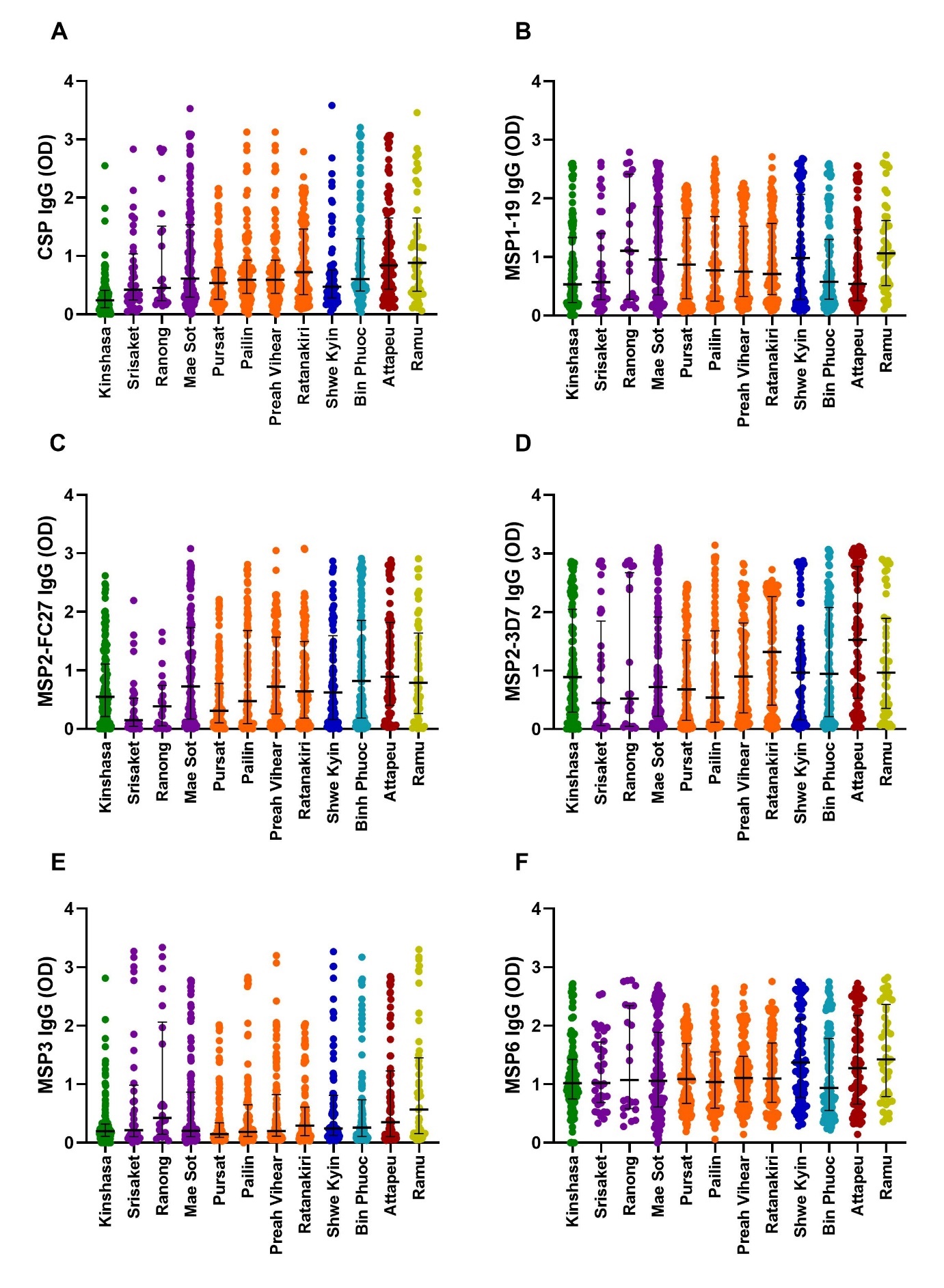

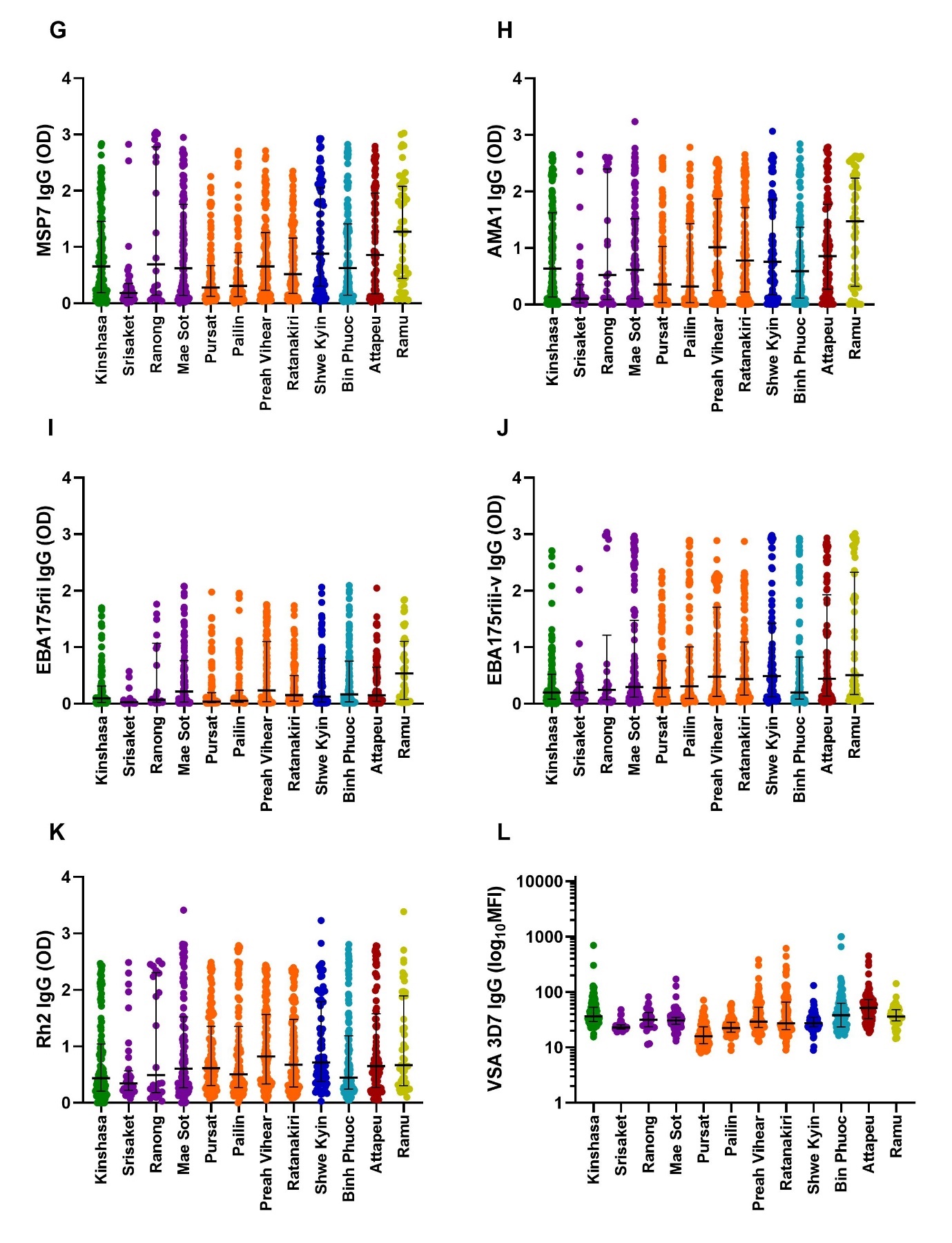


**Supplementary Figure 1: IgG responses to *P. falciparum* antigens in TRAC participants from DRC and Southeast Asian sites.** Dots represent individual IgG responses to selected recombinant *P. falciparum* antigens measured by ELISA (A-K) and variant surface antigens measured by flow cytometry (L). Black horizontal lines represent the median and whiskers represent the 25^th^ and 75^th^ percentiles. Sites are colour coded by country: DRC (green), Thailand (purple), Cambodia (orange), Myanmar (blue), Vietnam (cyan), Laos (dark red), Bangladesh (yellow). Seropositivity cut-offs for each antigen were as follows: CSP 0.40; MSP1-19 0.27; MSP2-FC27 0.03; MSP2-3D7 0.07; MSP3 0.14; MSP6 1.15; MSP7 0.17; AMA1 0.07; EBA175rii 0.11; EBA175riii-v 0.07; Rh2 0.30; VSA 3D7 336.1.

Supplementary Table 2: Seroprevalence of IgG responses to *P. falciparum* antigens in study participants

| **IgG response** | **n/N seropositive (%)** |  |  |  |  |  |  |  |  |  |  |  |
| --- | --- | --- | --- | --- | --- | --- | --- | --- | --- | --- | --- | --- |
|  | **Kinshasa, DRC (N=118)** | **Srisakhet, Thailand (N=36)** | **Ranong, Thailand** | **Mae Sot, Thailand** | **Pursat, Cambodia** | **Pailin, Cambodia**  **(N=96)** | **Preah Vihear, Cambodia** | **Ratanakiri, Cambodia**  **N=120** | **Shwe Kyin, Myanmar (N=77)** | **Bin Phuoc, Vietnam (N=118)** | **Attapeu, Laos** | **Ramu, Bangladesh (n=49)** |
| **Transmission stage antigen** |  |  |  |  |  |  |  |  |  |  |  |  |
| CSP | 32/118 (27.1) | 18/36 (50.0) | 12/22 (54.6) | 76/117 (65.0) | 68/119 (57.1) | 65/96 (67.7) | 83/120 (69.2) | 83/120 (69.2) | 43/77 (55.8) | 88/118 (74.6) | 66/85 (77.7) | 36/49 (73.5) |
| **Merozoite antigens** |  |  |  |  |  |  |  |  |  |  |  |  |
| MSP1_19_ | 82/118 (69.5) | 28/36 (77.8) | 17/22 (77.3) | 94/117 (80.3) | 90/119 (75.6) | 70/96 (72.9) | 98/120 (81.7) | 95/120 (79.2) | 59/77 (76.6) | 95/118 (80.5) | 69/85 (72.9) | 43/49 (87.8) |
| MSP2_FC27_ | 105/118 (89.0) | 29/36 (80.6) | 5/22 (77.3) | 109/117 (93.2) | 105/119 (88.2) | 87/96 (90.6) | 116/120 (96.7) | 111/120 (92.5) | 72/77 (93.5) | 110/118 (93.2) | 82/84 (97.6) | 44/49 (89.8) |
| MSP2_3D7_ | 104/118 (88.1) | 27/36 (75.0) | 16/22 (72.7) | 100/117 (85.5) | 102/119 (85.7) | 84/96 (87.5) | 116/120 (96.7) | 110/120 (91.7) | 69/77 (89.6) | 107/118 (90.7) | 81/84 (96.4) | 46/49 (93.9) |
| MSP3 | 80/118 (67.8) | 25/36 (69.4) | 17/22 (77.3) | 74/117 (63.3) | 63/119 (52.9) | 62/96 (64.6) | 80/120 (66.7) | 88/120 (73.3) | 55/77 (71.4) | 79/118 (67.0) | 59/85 (69.4) | 40/49 (81.6) |
| MSP6 | 49/118 (41.5) | 17/36 (47.2) | 11/22 (50.0) | 54/117 (46.15) | 50/119 (42.0) | 42/96 (43.75) | 57/120 (47.5) | 59/120 (49.2) | 47/77 (61.0) | 51/118 (43.2) | 47/85 (55.3) | 31/49 (63.3) |
| MSP7 | 91/118 (77.1) | 18/36 (50.0) | 15/22 (68.2) | 83/117 (70.9) | 75/119 (63.0) | 63/96 (65.6) | 96/120 (80.0) | 90/120 (75.0) | 64/77 (83.1) | 85/118 (72.0) | 64/85 (75.3) | 44/49 (89.8) |
| AMA1 | 94/118 (79.7) | 20/36 (55.6) | 17/22 (77.3) | 98/117 (83.8) | 86/119 (72.3) | 68/96 (70.8) | 101/120 (84.2) | 100/120 (83.3) | 66/77 (85.7) | 94/118 (79.7) | 77/85 (90.6) | 42/49 (85.7) |
| EBA175_Rii_ | 52/118 (44.1) | 3/36  (8.3) | 10/22 (45.5) | 67/117 (57.3) | 42/119 (35.3) | 32/96 (33.3) | 70/120 (58.3) | 64/120 (53.3) | 40/77 (52.0) | 69/118 (58.5) | 47/85 (55.3) | 35/49 (71.4) |
| EBA175_Riiiv_ | 93/118 (78.8) | 27/36 (75.0) | 17/22 (77.3) | 98/117 (83.8) | 100/119 (84.0) | 79/96 (82.3) | 110/120 (91.7) | 104/120 (86.7) | 69/77 (89.6) | 94/118 (79.7) | 74/85 (87.1) | 42/49 (85.7) |
| Rh2 | 78/118 (66.1) | 19/36 (52.8) | 14/22 (63.4) | 84/117 (71.8) | 90/119 (75.6) | 69/96 (71.9) | 100/120 (83.3) | 86/120 (71.7) | 66/77 (85.7) | 77/118 (65.3) | 62/84 (73.8) | 37/49 (75.5) |
| **Variant surface antigens** |  |  |  |  |  |  |  |  |  |  |  |  |
| VSA_3D7_ | 57/118 (48.3) | 8/36 (22.2) | 13/22 (59.1) | 37/117 (31.6) | 24/119 (20.2) | 17/96 (17.7) | 84/120 (70.0) | 62/120 (51.7) | 39/76 (51.3) | 74/118 (62.7) | 70/85 (82.4) | 29/49 (59.2) |

Supplementary Table 3. Multiple linear regression analyses of associations between antibody responses and PC_1/2_ in patients from Kinshasa DRC, adjusting for age and treatment

| **Antibody response** | **Coefficient** | **95% CI** | **P value** |
| --- | --- | --- | --- |
| **Transmission stage antigen** |  |  |  |
| CSP | -.04 | -.15, .07 | 0.505 |
| **Blood stage antigens** |  |  |  |
| MSP1_19_ | -.01 | -.10, .08 | 0.841 |
| PfMSP2_FC27_ | -.00 | -.08, .08 | 0.966 |
| PfMSP2_3D7_ | -.00 | -.08, .07 | 0.972 |
| MSP3 | .03 | -.08, .14 | 0.587 |
| MSP6 | -.02 | -.13, .08 | 0.656 |
| MSP7 | -.01 | -.10, .08 | 0.819 |
| AMA1 | -.02 | -.09, .06 | 0.627 |
| EBA175_Rii_ | .15 | .01, .29 | 0.040 |
| EBA175_Riiiv_ | .03 | -.06, .12 | 0.499 |
| Rh2 | .03 | -.06, .13 | 0.482 |
| **Variant surface antigens** |  |  |  |
| VSA_3D7*_ | -.07 | -.29, .15 | 0.536 |
|  |  |  |  |

Antibody responses were (log base 2(OD+0.01)) transformed, therefore, the coefficient represents the change in mean PC_1/2_ associated with a two-fold increase in antibody responses.
